# Anhydrous nanoprecipitation for the preparation of nanodispersions of tenofovir disoproxil fumarate in oils as candidate long-acting injectable depot formulations†

**DOI:** 10.1039/c9na00529c

**Published:** 2019-10-12

**Authors:** James J. Hobson, Paul Curley, Alison C. Savage, Amer Al-khouja, Marco Siccardi, Charles Flexner, Caren Freel Meyers, Andrew Owen, Steve P. Rannard

**Affiliations:** Department of Chemistry, University of Liverpool Crown Street Liverpool L69 7ZD UK srannard@liv.ac.uk; Department of Molecular and Clinical Pharmacology, University of Liverpool Block H, 70 Pembroke Place Liverpool L69 3GF UK; Department of Pharmacology and Molecular Sciences, The Johns Hopkins University School of Medicine 725 North Wolfe St. Baltimore MD 21205 USA; Department of Medicine, The Johns Hopkins University School of Medicine 575 Osler Building, 600 N. Wolfe St. Baltimore MD 21287 USA

## Abstract

The facile formation of drug nanoparticles in injectable/ingestible oils, of water-soluble antiretroviral tenofovir disoproxil fumarate, using a novel nanoprecipitation is presented with studies showing drug release into relevant aqueous media.

## Graphical abstract

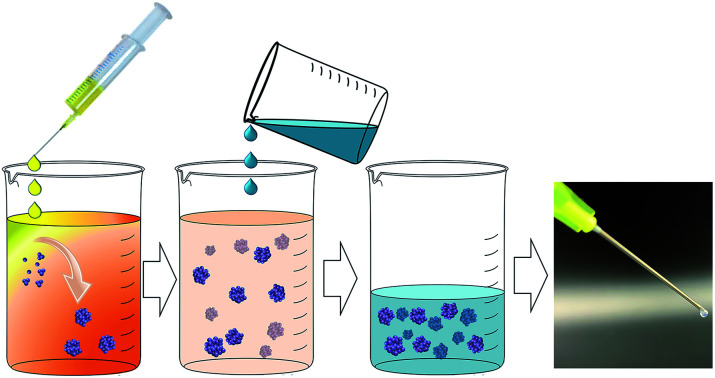

Traditionally, administration of drugs to humans for therapy or prophylaxis has been achieved primarily through oral dosing. For regimens that require repeated dosing over very long timescales, daily oral doses of tablets, capsules or liquids serve as a reminder of disease and erode quality of life.^1^ This problem is particularly prevalent in chronic diseases such as human immunodeficiency virus (HIV) where non-adherence to therapy is a major contributor to the rise in drug-resistant virus transmission and ultimate failure of therapeutic options.^2^

Over the last 50 years, the use of long-acting depot injections (LAIs), delivered subcutaneously or intramuscularly, has improved patient adherence, clinical outcomes and maintenance of chronic conditions including schizophrenia, alcohol dependence, androgen ablation and contraception.^3^ In recent years, the benefits of LAIs for HIV have been demonstrated in therapy and pre-exposure prophylaxis. These formulations comprise nano-milled dispersions^4^ of poorly water-soluble antiretrovirals (*e.g.* cabotegravir or rilpivirine)^5,6^ that are intramuscularly administered and provide systemic drug exposure for >1 month; in the case of cabotegravir, up to three months of drug exposure within the therapeutic window has been achieved.

LAIs for diseases such as HIV can change a patient's life by offering a small number of regular injections rather than daily combinations of different drug classes. There is, however, the need for combination therapies within HIV management that has added to the complexity for LAI approaches. Typically, HIV requires a minimum of two nucleoside reverse transcriptase inhibitors (NRTIs) plus an additional antiretroviral drug such as a protease inhibitor.^7^ The NRTI “backbone” drugs are water-soluble and, therefore, are not readily compatible with conventional nano-milling; depot injections of cabotegravir or rilpivirine would require daily supplementation of orally dosed NRTI tablets which effectively negates the benefits of their LAI formulations. Bimonthly rilpivirine/cabotegravir combination injections are in late stage development and have shown to be as effective as orally dosed combinations of cabotegravir, abacavir and lamivudine in suppression of HIV-1 viral replication;^8^ however, two-drug combinations are not currently a standard of care. Unfortunately, water-soluble NRTIs such as emtricitabine, tenofovir disoproxil fumarate (TDF), lamivudine and abacavir are not currently available as LAI formulations to combine with ongoing clinical opportunities such as the poorly soluble cabotegravir (integrase inhibitor) or rilpivirine (non-nucleoside reverse transcriptase inhibitor).

The formation of LAI formulations from water-soluble drugs is more challenging without significant chemical modification or added materials to alter solubility; both strategies lead to increased injection volumes to achieve adequate drug dosing. Injections of aqueous solutions may lead to very high local drug concentrations and “dose dumping” can lead to highly attenuated drug delivery durations. The formation of hydrophobic prodrugs, that are activated to parent compounds (*i.e.* release the desired drug compound) *in vivo*, has enabled injectable oil-solutions^9^ to be administered clinically and nano-milled dispersions to be formed in aqueous media when relatively high melting point solid prodrugs are synthesised;^10^ recently we have shown the benefits of long acting aqueous nanoparticulate antimalarial depot injections to prevent malarial infection in pre-clinical species^11^ and the use of emulsion templated freeze drying (ETFD)^12,13^ to form semi-solid prodrug nanoparticles where the resulting prodrugs have low melting points.^14^

Here, we investigate a novel approach of LAI formulation applied directly to TDF, a prodrug of the parent NRTI tenofovir (TFV; Fig. 1A). The potential for nano-dispersion formation in oils, avoiding prodrug strategies and attrition processes such as non-aqueous nano-milling or high-pressure homogenisation, has been investigated with the target of producing injectable-oil formulations containing dispersed water-soluble drug nanoparticles.

**Fig. 1:**
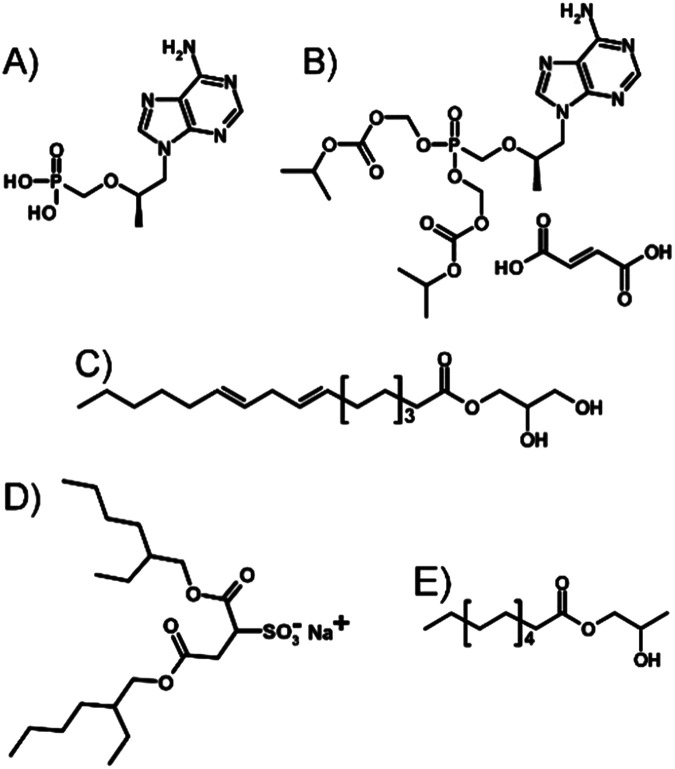
Chemical structures of (A) tenofovir (TFV), (B) tenofovir disoproxil fumarate (TDF), (C) glycerol monolinoleate (Maisine 35-1; M 35-1), (D) sodium bis(2-ethylhexyl) sulfosuccinate (AOT), and (E) propylene glycol monolaurate (Lauroglycol FCC; LG FCC).

Low-energy formation of nanoparticles has been successfully achieved by nanoprecipitation processes for many years.^15^ In general, a water-miscible organic solvent is utilised to dissolve poorly water-soluble compounds (such as linear^16^ or branched polymers^17,18^ and active pharmaceutical ingredients),^19,20^ and controlled addition to water results in a dilution of the water-miscible “good” solvent regime and the formation of a “bad” solvent, or antisolvent, environment. During this process, dissolved materials nucleate, precipitate and form colloidal structures; in the presence of appropriate stabilisation, macro-phase separation may be avoided, and colloidal stability can be induced whilst the particles are still within the nanoscale. This has been successfully exploited at scale to form a range of products including β-carotene nanoparticles for health, food and veterinarian products.^21^ Recent reports of “inverse nanoprecipitation”^22^ have utilised the addition of aqueous solutions into water-miscible organic solvents, such as acetone, with subsequent crosslinking leading to hydrophilic nanoparticles. “Inverse flash nanoprecipitation” to encapsulate biologics has also been reported; in this process, an aqueous/solvent mixture (*e.g.* dimethyl sulfoxide) of the biologic and a charged amphiphilic block copolymer is rapidly mixed with an antisolvent such as dichloromethane (DCM) to precipitate the hydrophilic biologic with stabilisation derived from the hydrophobic segments of the block copolymer.^23^ To the best of our knowledge, no reported nanoprecipitations of organic compounds are completely anhydrous, and all of them employ water in either the solvent or antisolvent phase.

Several key considerations must be addressed to achieve the nanoprecipitation of water-soluble antiretroviral salts such as TDF (Fig. 1B) using anhydrous liquid media and resulting in an injectable oil dispersion. Ideally, stabilisers that prevent macroscale phase-separation are solvent, antisolvent and injectable oil soluble; TDF must be insoluble in the chosen injectable oil but soluble within a solvent which is miscible with an antisolvent for the drug substance.

TDF was found to be soluble in methanol (MeOH) to a concentration of >80 mg mL^−1^ and found to be substantially insoluble in peanut oil, sesame oil, soy bean oil and castor oil; sesame and peanut oils were selected due to their widespread use in depot injections and to introduce diversity of oil chemistry to this study. Dichloromethane was selected as the antisolvent due to its good miscibility with methanol, and a screening approach was used to study 52 potential commercial lipophilic stabilisers, such as glycerol monolinoleate (commercially known as Maisine 35-1 (M 35-1), Fig. 1 C) and propylene glycol monolaurate (commercially known as Lauroglycol FCC (LG FCC), Fig. 1E); 18 candidate stabilisers were determined as having an appropriate solubility profile described above (ESI Table S1†). Within the identified stabilisers, a series of non-ionic molecules were identified and one anionic surfactant, namely sodium bis(2-ethylhexyl)sulfosuccinate, was also commercially known as docusate sodium or Aerosol OT® (AOT, Fig. 1D). Organic sulfonates are known to ion-exchange with ammonium salts, in particular halides, to generate ammonium sulfonates with relatively low solubilities; indeed, this simple aqueous reaction has been reported in the formation of suspensions of ionic chiral polymers.^24^ The form 1 crystal structure of TDF shows an interaction of the protonated amino functionality with the associated fumarate, with significant changes occurring on heating.^25^ The potential for ion-exchange reactions to drive nanoprecipitation under anhydrous conditions was therefore studied in an attempt to establish conditions for precipitation during ion-exchange of TDF and AOT. As with recent reports of isothermal titration calorimetry (ITC) of aqueous mixtures of ammonium and sulfonate surfactants,^26^ complex formation was observed between AOT and TDF in water, with subsequent nanoparticle formation as determined by dynamic light scattering (DLS). After full titration of the interaction, a particle dispersion with a *z*-average diameter (*D*_*z*_) of approximately 270 nm was observed (Fig. 2).

**Fig. 2:**
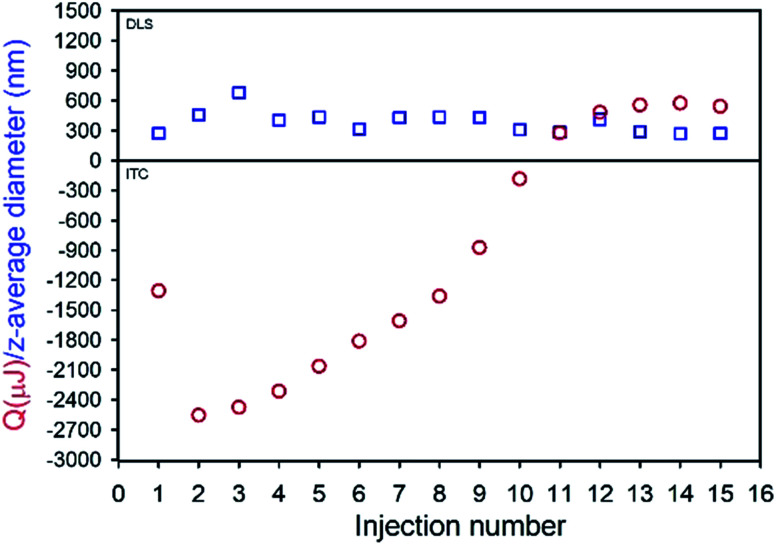
Isothermal titration calorimetry (red circles) and corresponding dynamic light scattering (blue squares) analyses of AOT + TDF titration in water.

The ITC studies were highly reproducible and clearly indicated interactions occurring during mixing of AOT and TDF in water; however, model fitting (ESI Fig. S1†) to determine stoichiometry of the interaction^27^ suggested a multiple site mechanism and was possibly complicated by the measured enthalpies being derived from simultaneous complexation and nanoprecipitation.

Having observed nanoparticle formation in water, a strategy for nanoprecipitating TDF in the presence of AOT under anhydrous, but polar, solvent conditions and readily resulting in an injectable oil dispersion of the water-soluble NRTI was devised (Fig. 3). TDF was dissolved in MeOH and added slowly to a DCM solution of AOT and optional additional lipophilic surfactants identified during the solubility screening described earlier. Displacement of the fumarate counterion by AOT (Fig. 3A(ii)), led to the formation of a mixed DCM/MeOH dispersion (Fig. 3A(iii)). Control experiments, carried out in the absence of AOT, showed no stable nanoparticle formation (ESI Fig. S2†). Within the samples that successfully created dispersions (ESI Fig. S3–S11†), AOT was shown to work independently and in combination with several secondary stabilisers; M 35-1 and LG FCC were selected for further study due to dispersion reproducibility and apparent quality. The organic dispersion (DCM/MeOH) is a good solvent for the selected peanut and sesame oils which are, in turn, poor solvents for TDF as identified through earlier screening. Addition of varying volumes of these oils to the organic dispersion (Fig. 3B(i)) led to a combined injectable oil/DCM/MeOH dispersion (Fig. 3B(ii) and C(i)). The dispersion was frozen using cryogenic liquids and subjected to freeze drying to remove all volatile components of the mixture (Fig. 3B(iii) and C(ii, iii)). The process allows direct control of drug concentration and ultimate injected dose through simple modification of injectable oil volumes prior to freeze drying. It also avoids well reported issues of redispersion of freeze-dried suspensions and dispersions, as the nanoparticles are not dried and redispersed but, rather, a liquid oil suspension is generated directly in the single freeze-drying step. Loading into syringes was readily accomplished (Fig. 3C(iv)); the critical consideration of “syringeability”^28^ was evaluated using a 21 gauge needle (Fig. 3C(v)) and oil dispersions with TDF concentrations of up to 60 mg mL^−1^ (TDF loadings up to 80 wt% relative to AOT and lipophilic surfactants).

**Fig. 3:**
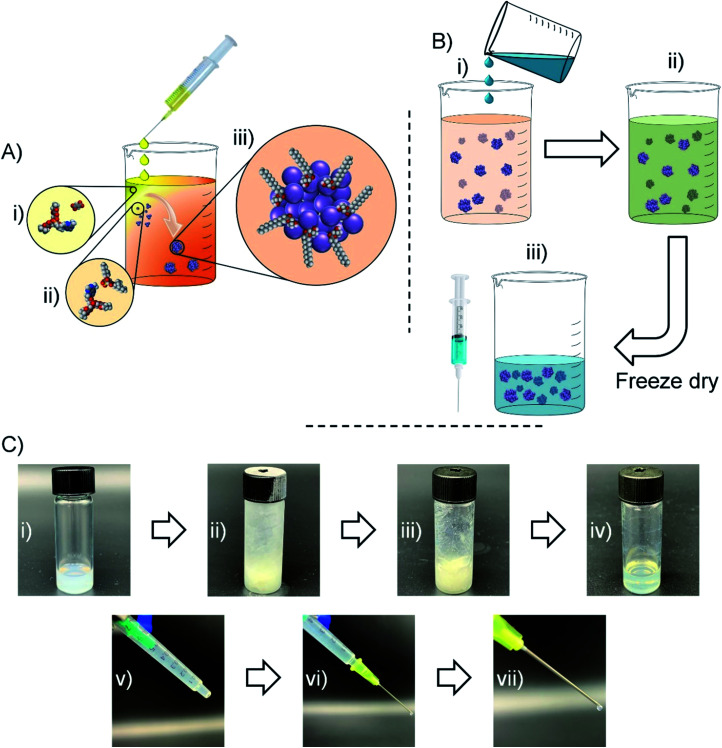
Strategy for formation of injectable oil formulations: (A) addition of a methanol solution of TDF (i) to a DCM solution of AOT (and optional additional stabilisers) with subsequent complexation (ii) and nanoprecipitate formation (iii); (B) addition of injectable oil to the MeOH/DCM mixture containing nanoprecipitates (i) and formation of a final mixed liquid dispersion (ii) with freezing and freeze-drying to leave a dispersion of nanoprecipitates in oil (iii). Photographs of the steps: (C) (i) nanoprecipitate dispersion containing solvents and oil; (ii) frozen dispersion; (iii) frozen dispersion after freeze-drying; (iv) final dispersion in oil after melting; (v) loading into syringe (without needle); (vi and vii) dispensing of the nanoprecipitate dispersion in oil.

The DCM/MeOH nanoprecipitation process was initially studied by DLS, with samples analysed prior to oil addition and without freezing (Table 1). This simple study clearly showed that in the absence of AOT, LG FCC/M 35-1 mixtures were unable to create stable drug nanoparticles. AOT, and AOT mixtures with LG FCC or M 35-1 formed narrow nanoparticle dispersions with *D*_*z*_ values of approximately 540 nm (ESI Fig. S3–S5†). Dynamic light scattering analysis of nanoparticles in viscous oil media is not straightforward; therefore samples were diluted after freeze-drying using DCM to a TDF concentration of 0.05 mg mL^−1^ and DLS studies were conducted to determine *D*_*z*_ and polydispersity (PDI). With the caveat that these values are potentially not fully representative, the presence of the different oils appeared to have a relatively minor influence on the observed *D*_*z*_ values, even though nanoparticle formation occurs prior to oil addition (ESI Fig. S6–S11†). Sesame oil and peanut oil are chemically dissimilar, despite essentially comprising considerable fractions of fatty acid glycerides derived from linoleic and oleic acids;^29–31^ sesame oil is also known to contain a significant fraction of non-fatty acid derived compounds including polyphenols (lignans) such as sesamin, sesamolin, episesamin, sesamol and sesaminol (Fig. 4).^32^

**Table 1:** Dynamic light scattering and dialysis release studies of varying TDF nanoprecipitates

Stabiliser	DLS^a^		Dialysis (total NRTI)^b^	
	*D*_*z*_ (nm)	PDI	Release rate (h^−1^)	Release^c^ (%; 6 h)
**No oil**				
LG FCC/M 35-1^d^	*5874*	*0.780*	—	—
AOT	540	0.164	—	—
AOT/LG FCC	545	0.148	—	—
AOT/M 35-1	540	0.177	—	—

**Sesame oil**				
AOT	295	0.373	0.0098	8.3 ± 2.7
AOT/LG FCC	512	0.242	0.0104	7.7 ± 0.4
AOT/M 35-1	584	0.343	0.0126	8.7 ± 1.8

**Peanut oil**				
AOT	672	0.456	0.0060	4.8 ± 1.4
AOT/LG FCC	840	0.383	0.0119	9.0 ± 0.7
AOT/M 35-1	815	0.327	0.0060	4.4 ± 1.6

a 1 mg mL^−1^ TDF dispersed in DCM; quartz crystal cuvette.

b Spectra/Por® Float-A-Lyzer® G2 chamber (100 kDa MWCO).

c Cumulative release of total NRTI (*n* = 3).

d Poor data quality reported for completeness. See Table S2 for the complete dataset.

**Fig. 4:**
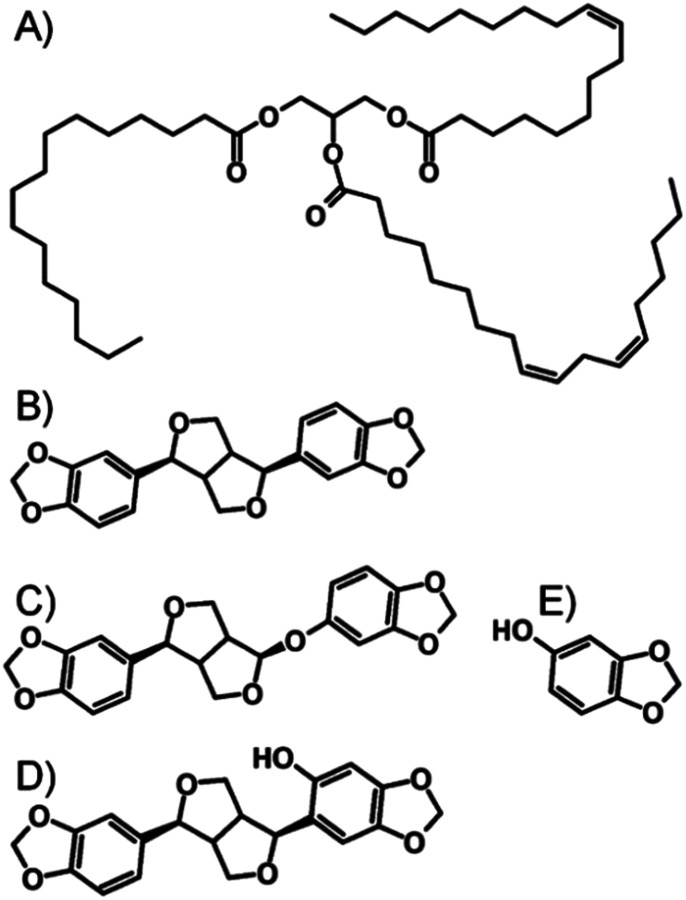
Chemical structures of (A) peanut oil, and non-lipid components of sesame oil, namely (B) sesamin, (C) sesamolin, (D) sesaminol and (E) sesamol.

The complexity of the sesame oil environment appeared to have little impact on the nanoparticle dispersions, with similar *D*_*z*_ values observed for nanoparticles formed in the presence of AOT/LG FCC and AOT/M 35-1; slightly smaller values were seen when AOT was utilised solely (Table 1). As ion exchange appears to dictate insolubility and the drug/AOT complex presents a polar substrate for adsorption of the lipophilic stabilisers, which all contain hydroxyl functionality, it seems reasonable to suggest that the stabilisers are adsorbed through processes such as electrostatic or H-bonding interactions and the lipophilic environments are unable to perturb the stabilising molecules.

Nanoparticles measured in the presence of peanut oil are noticeably larger (Table 1), suggesting additional association of components of peanut oil with the stabilised drug/AOT complex; further dilution using DCM does not liberate nanoparticles that are similar in size to either those diluted from sesame oil or those measured in MeOH/DCM directly after formation.

Release of prodrugs from intramuscular oil-solution depot injections is well established clinically; however, release of a water-soluble drug from these candidate long-acting depot formulations must be determined to evaluate potential future value. The six oil dispersions (Table 1) were, therefore, subjected to dialysis studies using Spectra/Por® Float-A-Lyzer® G2 dialysis chambers (molecular weight cut off = 100 kDa) submerged in simulated interstitial fluid (see the ESI†) to model conditions within the muscle environment. The release of TDF and parent TFV (after hydrolysis) was able to be monitored individually at each time point (Fig. 5A), to allow calculation of total cumulative NRTI drug release over 6 hours utilising liquid chromatography-mass spectrometric analysis (Fig. 5B; see the ESI†). Long-acting therapeutics require extended drug release, aiming to present patients with therapeutic exposures for weeks/months after administration. The model dialysis experiments conducted here have been validated in previous studies with different drug compounds to correlate well with *in vivo* behaviour^11,33^ and all show the appearance of the water-soluble parent TFV and the TDF prodrug within the simulated interstitial fluid over prolonged periods.

**Fig. 5:**
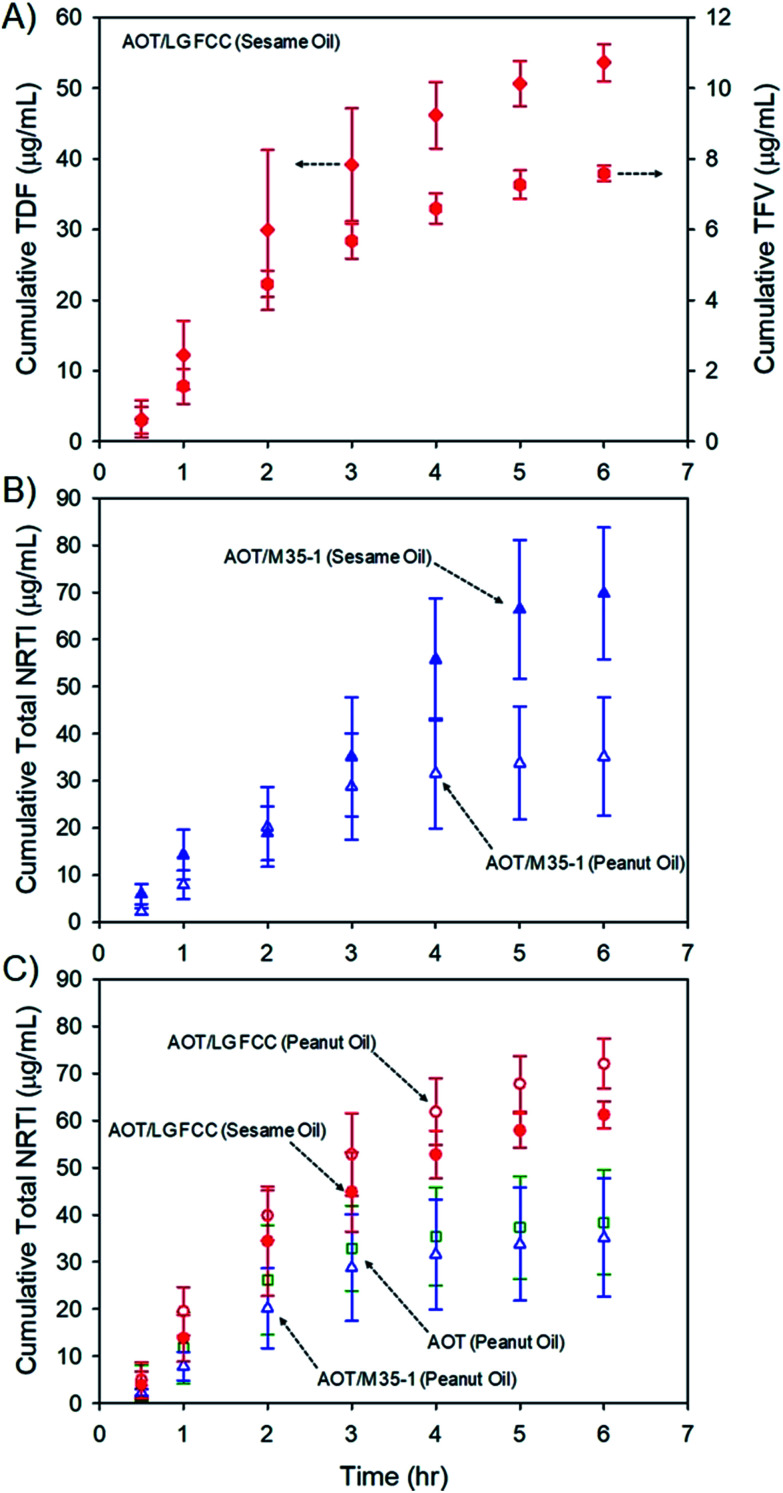
NRTI release studies from nanoprecipitate dispersions in oils. (A) example of individual quantification of TDF (diamonds) and TFV release (circles) from sesame oil; (B) example of cumulative NRTI (TDF + TFV) release differences from dispersions containing peanut (open triangles) or sesame oils (closed triangles); and (C) overlay comparison of cumulative NRTI release from varying stabiliser mixtures in peanut oil (open symbols) and a comparative sesame oil release curve (closed circles).

The rates of release cannot be directly related to behaviour in humans as, despite previous validation against clinical data, the dialysis methodology was developed for hydrophobic drug particles dispersed in water and specific *in vivo* evaluation is now required. Additionally no current clinical product exists to derive comparisons within this model; however, several observations may be made: first, the presence of TFV was consistently higher in all cases where sesame oil was used as the vehicle (ESI Fig. S12†); second, total NRTI drug release rates appear to be faster from sesame oil environments (ESI Fig. S13†), although samples comprising Lauroglycol FCC and AOT showed similar release rates from the different oils over the time period studied (Fig. 5C) and, finally, the release of total NRTI from sesame oil samples appears to be relatively independent of stabiliser composition. As mentioned earlier, the composition of peanut and sesame oils varies considerably, and although the impact of these materials on the adsorption, or dynamic equilibrium, of lipophilic stabilisers, or the saturation solubility of TDF complexes, is not known, the marked difference in drug release from the different hydrophobic environments does suggest an opportunity for moderate release kinetics. It is also important to note that trough plasma concentration values of TFV in patients undertaking daily oral dosing are approximately 40 ng mL^−1^ which would act as a minimum target circulating drug concentration to maintain levels within the therapeutic window for extended periods;^34^ prolonged concentrations of approximately 80 ng mL^−1^ in plasma are associated with renal toxicity.^35^

In conclusion, a considerable clinical need to adopt long-acting therapeutics within global HIV management exists; however, the lack of a wide range of long-acting candidates which encompass well established drug classes, such as NRTIs, may delay full realisation of patient benefits and the creation of a pipeline of combination options for therapy. Water-soluble drugs are not readily converted to long-acting depot formulations without either prodrug approaches, polymer-controlled release strategies (*e.g.* microspheres or *in situ*-forming depot formulations), or the use of oil carriers. The approach presented here offers a rapid and facile method to create injectable oil dispersions that release TDF. This is a critical drug that is widely used orally across the world and systemic pharmacokinetics and pharmacodynamics have been clinically established. As such, repurposing to long acting depot injections has a clear development and regulatory pathway. Importantly, the strategy presented here creates a new administration format without the need for new chemical entity synthesis and the clinical and financial complexities this would create. Additionally, sterile freeze drying^36^ (often termed lyophilisation) is a well-known and common manufacturing process for injectable products (including vaccines) and the approach here should be able to be scaled using standard procedures as a wide range of solvents can be removed using freeze-drying to prescribed acceptable limits.^37^ Many mechanisms may dictate the actual observed release rates *in vivo*, including the formation of a granuloma after macrophage infiltration,^38^ and “flip-flop” kinetics (circulating drug concentrations determined by release from the injection site and not clearance/elimination mechanisms) that have been observed in antipsychotic LA medication.^39^ We understand that this demonstration is the first step towards the establishment of clinical value and further work is required, including determination of appropriate *in vivo* release kinetics, studying long term stability, tolerability, *in vivo* pharmacokinetics and injection site toxicology; however, we have demonstrated a route which may unlock future developments and stimulate the progression of novel long acting NRTI therapies.

## Conflicts of interest

The authors are inventors on patents that seek to protect TDF nanoprecipitation to allow charitable access. C. F. reports serving as a paid consultant for Cipla Pharmaceuticals, Janssen Pharmaceuticals, Merck Laboratories, Mylan Pharmaceuticals, and ViiV Healthcare, and received research grant support from Gilead Sciences paid to his University. AO and SR are directors of Tandem Nano Ltd. AO and SR have received research funding from ViiV Healthcare and AstraZeneca; AO has acted as a personal consultant to Gilead, ViiV Healthcare and Merck. The authors declare no competing interests.

## Supplementary Material

NA-001-C9NA00529C-s001
