# Supplementary material for: Anhydrous nanoprecipitation for the preparation of nanodispersions of tenofovir disoproxil fumarate in oils as candidate long-acting injectable depot formulations
Source: Nanoscale Adv. 2019 Oct 12;1(11):4301–7. doi: 10.1039/c9na00529c (PMC9417103; doi:10.1039/c9na00529c)
Supplement: NA-001-C9NA00529C-s001 [file NA-001-C9NA00529C-s001.pdf]

Electronic Supplementary Material (ESI) for Nanoscale Advances.
This journal is © The Royal Society of Chemistry 2019

# Anhydrous nanoprecipitation for the preparation of nanodispersions of tenofovir disoproxil fumarate in oils as candidate long-acting injectable depot formulations

James J. Hobson,[a] Paul Curley,[b] Alison C. Savage,[a] Amer Al-khouja,[c] Marco Siccardi,[b] Charles Flexner, [c,d] Caren Freel Meyers,[c] Andrew Owen[b] and Steve P. Rannard*[a]

[a.] *Department of Chemistry, University of Liverpool, Crown Street, Liverpool L69 7ZD, UK. Email: srannard@liv.ac.uk*
[b.] *Department of Molecular and Clinical Pharmacology, University of Liverpool, Block H, 70 Pembroke Place, Liverpool L69 3GF, UK.*
[c.] *Department of Pharmacology and Molecular Sciences, The Johns Hopkins University School of Medicine, 725 North Wolfe St., Baltimore, MD 21205, USA.*
[d.] *Department of Medicine, The Johns Hopkins University School of Medicine, 575 Osler Building, 600 N. Wolfe St., Baltimore, MD 21287, USA.*

Email:srannard@liverpool.ac.uk

# Supplementary Information

## Materials

Castor oil, peanut oil, soy bean oil, sesame oil, AOT, Brij™ S2, Span™ 80, Tween™ 40, Tween™ 80, phosphate buffered saline, fetal bovine serum, were all purchased from Sigma-Aldrich (Dorset, UK). Dichloromethane, acetonitrile, and methanol were bought from Fisher Scientific (Loughborough, UK). Tenofovir disoproxil fumarate was supplied by Laurus Labs (Parawada, India), and Capryol™ 90, Capryol™ PGMC, Labrafac™ PG, Labrafil™ M 1944 CS, Labrafil™ M 2125 CS, Labrasol™, Lauroglycol™ 90, Lauroglycol™ FCC, Maisine™ 35-1, Monosteol™, Pecol™, Plurol™ diisosteraque, Plurol™ Olique, and were supplied by Gattefosse (Saint-Priest, France).

## Methods

### Generation of tenofovir disoproxil fumerate nanosuspension

In a typical experiment to synthesise a 1 mL total volume of 80 wt% nanoprecipitated TDF particles the following procedure was used. Firstly, stock solutions of tenofovir disoproxil fumarate (TDF) and stabilisers were made. For the stabilisers, 5 mg/mL of the relevant stabiliser was dissolved in dichloromethane (DCM) and left to roll continuously until fully dissolved. 80 mg/mL of TDF was dissolved in methanol (MeOH) by using a magnetic stirrer bar and magnetic stirring plate at ambient temperature. Stock solutions of TDF in MeOH had to be used as soon as dissolved and could not be kept for synthesis on subsequent days as TDF is a pro drug of tenofovir, and as such would undergo hydrolysis if stored as a solution in MeOH. 400 µL of 5 mg/mL of AOT dissolved in DCM was pipetted into a 4 mL volume glass vial, followed by 400 µL of 5 mg/mL Maisine 35-1 or Lauroglycol FCC, dissolved in into the same vial. 200 µL of 80 mg/mL TDF dissolved in MeOH was then rapidly added to the same vial, and the vial briefly shook. It was observed that upon shaking a white hazy nanoprecipitate was formed instantaneously.

To produce the final dosage form, the oil phase was pipetted directly into the nanoprecipitated TDF particles. The oils were miscible with the MeOH/DCM nanoprecipitation, and as such could be homogeneously mixed by vortexing. This mixture was then rapidly frozen using liquid nitrogen, and subsequently underwent 48 hours of freeze drying in a VirTis Benchtop K freeze dryer, with condenser set to -100°C and pressure at >20 µBar. The freeze-drying procedure removed both the MeOH and the DCM phases from the mixture, leaving behind the TDF particles dispersed in the oil phase, which is initially frozen, but after ~5 minutes was observed to melt, ready for use in any subsequent *in vitro* or *in vivo* testing. The concentration and volume of the final particle-in-oil suspension could be finely tuned by simply altering the quantities of nanoprecipitate and oil combined prior to freeze drying. High concentrations (60 mg/mL) of particles in oil have been achieved, with the particles forming a thick suspension in the oil. This suspension could be taken up into a syringe and passed back through a 25G needle despite the high viscosity, allowing for use as an injectable dose. Similarly, low concentrations can also be achieved for use in sensitive *in vitro* assays, producing clearer nanoparticle dispersions.

### Characterisation

Dynamic Light Scattering (DLS) was used to determine droplet diameter and surface charge using a Malvern ZetaSizer Nano ZS instrument. Samples were measured in plastic zeta cells,

which allowed for measurement of both z-average diameter and ζ-potential. Samples were diluted to obtain a laser attenuation of 5 or 6 and measured 3 times for both diameter and surface charge, with each measurement having an automated number of scans, as determined by the Zetasizer software. The average value from the 3 scans was reported and the temperature within the measurement cell was set to a constant 25 ºC.

**Isothermal Titration Calorimetry**

Isothermal titration calorimetry was performed using a NanoITC instrument supplied by TA Instruments (Elstree, UK). The sample cell contained a solution of 1 mg/mL of TDF dissolved in water, whilst the sample syringe contained a solution of 10 mg/mL AOT dissolved in water. The reference cell contained water, with the experiment carried out at a fixed temperature of 25$^{o}$C. The needle contained 250 µL of AOT solution, which was titrated into the sample cell containing TDF solution in 15 equal injections of 15 µL. The interval time between injections was fixed at 300 seconds and the stirring was set to 250 RPM. The heat change was recorded and the enthalpy of dilution of the TDF solution was subtracted from the raw data. Data analysis was performed using the NanoAnalyze software (TA Instruments, Elstree, UK), and fitted using the multiple sites model.

***In vitro* release Dialysis**

The *in vitro* release rate of candidate formulations from dialysis chambers (Spectra-Port Float-A-Lyzer® G2 dialysis tubes MWCO 100kD) was determined over 6 hours. 1mL of test formulation (10mg/mL SDN, 80% active pharmaceutical ingredient [API]) was loaded into dialysis chambers and placed in 50mL tubes containing 15mL of simulated interstitial fluid (SIF, 50% PBS 50% FBS). The tubes were incubated at 37$^{o}$C on a rotating manifold. At 30 mins, 1h, 2h, 3h, 4h, 5h and 6h, the dialysis tubes were removed from the SIF and placed in a new 50mL tube containing fresh SIF. Samples were aliquoted and stored at -80$^{o}$C until analysis via LCMS. The cumulative amount released from each candidate was plotted in order to determine the rate of release.

**Liquid chromatography Mass spectrometry analysis**

Quantification was achieved via LC-MS/MS (TSQ Endura, Thermo Scientific) operating in positive mode. The following ions were monitored for quantification in selected reaction monitoring scan: TFV (m/z 288.1 > 159.1, 176.0 and 270) and TDF (m/z 520.1 > 270, 288.1 and 300). A stock solution of 1 mg/mL was prepared in $H_2O$ and stored at 4°C until use. A standard curve was prepared in SIF by serial dilution from 500 ng/mL to 1.9 ng/mL and an additional blank solution was also used. Extraction was achieved via protein precipitation. Samples were diluted with ACN (sample: ACN ratio = 1:1) and thoroughly vortexed. Samples were then centrifuged at 4000 × g for 10 min at 4°C. The supernatant fraction was transferred to a fresh glass vial and evaporated; samples were placed in a rotary vacuum centrifuge at 30°C and then reconstituted in 140 µL of $H_2O$:ACN (95:5). 100 µL of the sample was then transferred into 200 µL chromatography vials.

Chromatographic separation was achieved using a multi-step gradient with a Hypersil gold aQ C-18 column (Thermo scientific) using mobile phases A (100% $H_2O$, 0.1% formic acid) and B (100% ACN, 0.1% formic acid). Chromatography was conducted over 9 minutes at a flow rate of 300 µL/min. At the start of each run, mobile phase A was 100% for 1 minute when mobile phase B was increased to 86% at 1.5 minutes. Mobile phase B was

then gradually increased to 92% over 5.5 minutes. Mobile phase B was then increased to 97% at 6.5 minutes which was held until 7.5 minutes. Mobile phase A was then increased to 100% and held till the termination of the run at 9 minutes. Inter- and intra-day accuracy and precision was assessed using spiked quality controls at 20 ng/mL, 100 ng/mL and 400 ng/mL. Inter- and intra-day accuracy and precision were within 20% for both TFV and TDF.

Table S1. List of excipients selected from screening protocol based on HLB values, solubility in DCM, and solubility in oil continuous phase

| **Final Excipient Selection** |
|---|
| Brij™ S20 |
| AOT |
| Capryol™ 90 |
| Capryol™ PGMC |
| Labrafac™ PG |
| Labrafil™ M 1944 CS |
| Labrafil™ M 2125 CS |
| Labrasol™ |
| Lauroglycol™ 90 |
| Lauroglycol™ FCC |
| Maisine™ 35-1 |
| Monosteol™ |
| Pecol™ |
| Plurol™ diisosteraque |
| Plurol™ Olique |
| Span™ 80 |
| Tween™ 40 |
| Tween™ 80 |

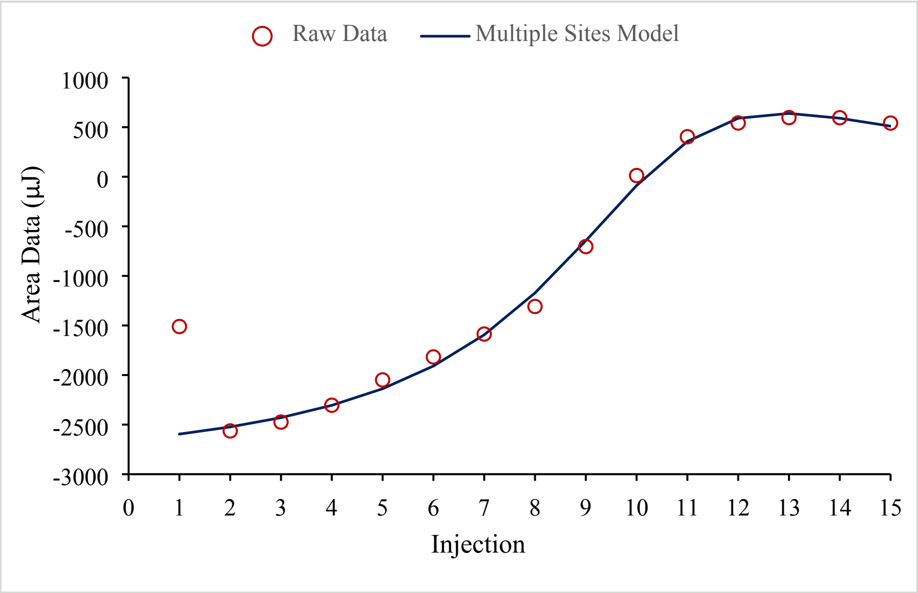


| Multiple Sites Model | |
|---|---|
| Ka1 (M$^{-1}$) | 9.392 x $10^{3}$ |
| Ka2 (M$^{-1}$) | 1.170 x $10^{3}$ |
| n1 | 2.468 |
| n2 | 0.100 |
| ΔH1 (kJ/mol) | -8.751 |
| ΔH2 (kJ/mol) | 156.2 |
| Kd1 (M) | 1.065 x $10^{-4}$ |
| Kd2 (M) | 8.545 x $10^{-4}$ |
| ΔS1 (J/mol.K) | 46.71 |
| ΔS2 (J/mol.K) | 582.6 |

Figure S1. Multiple site model fitting for the isothermal titration calorimetry analysis of TDF/AOT nanoprecipitation in water.

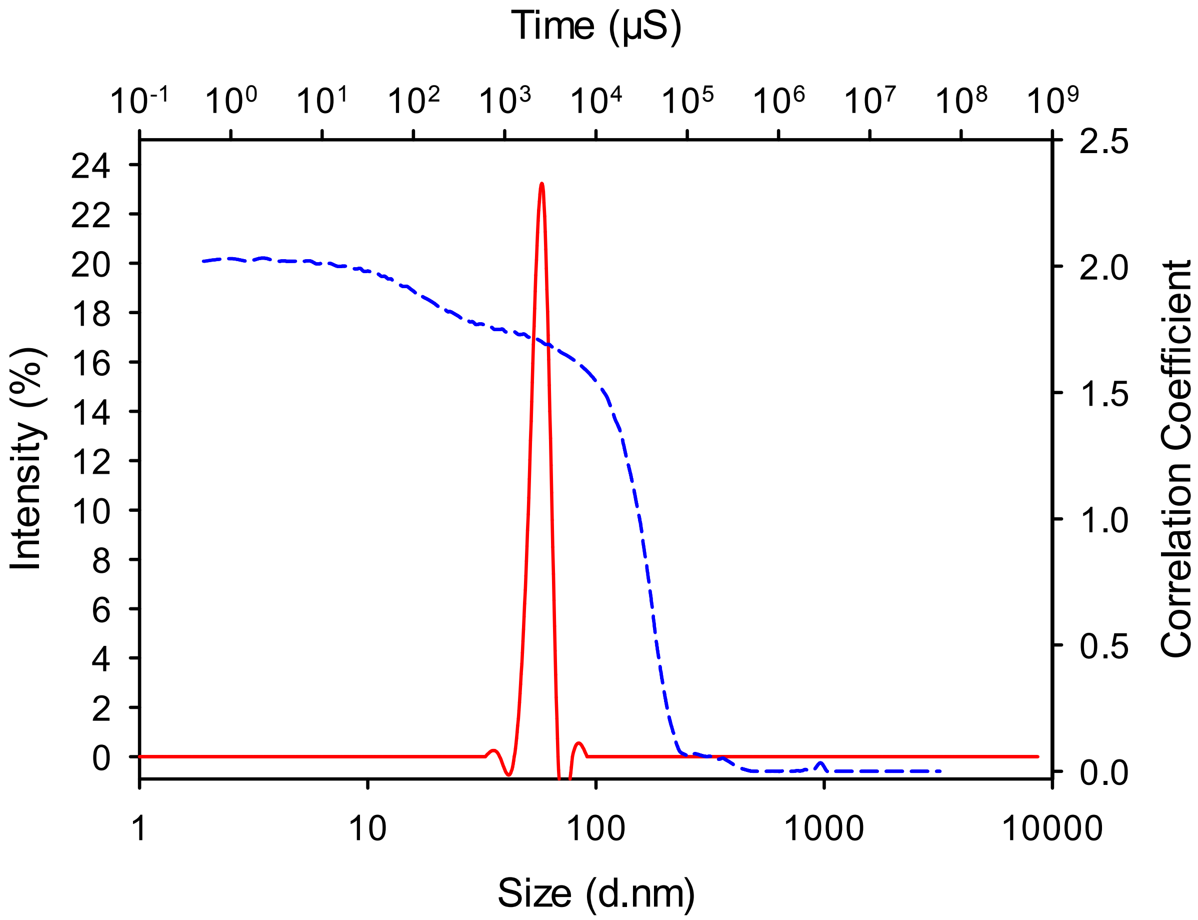


Figure S2. Size distribution and correlation coefficient of TDF precipitated with Lauroglycol FCC and Maisine 35-1 and measured in DCM continuous phase (no AOT present). Poor quality data is generated, as shown by the non-sigmoidal appearance of the correlation coefficient. Measurements are an average of 3 scans using a Malvern Zetasizer Nano S dynamic light scattering instrument.

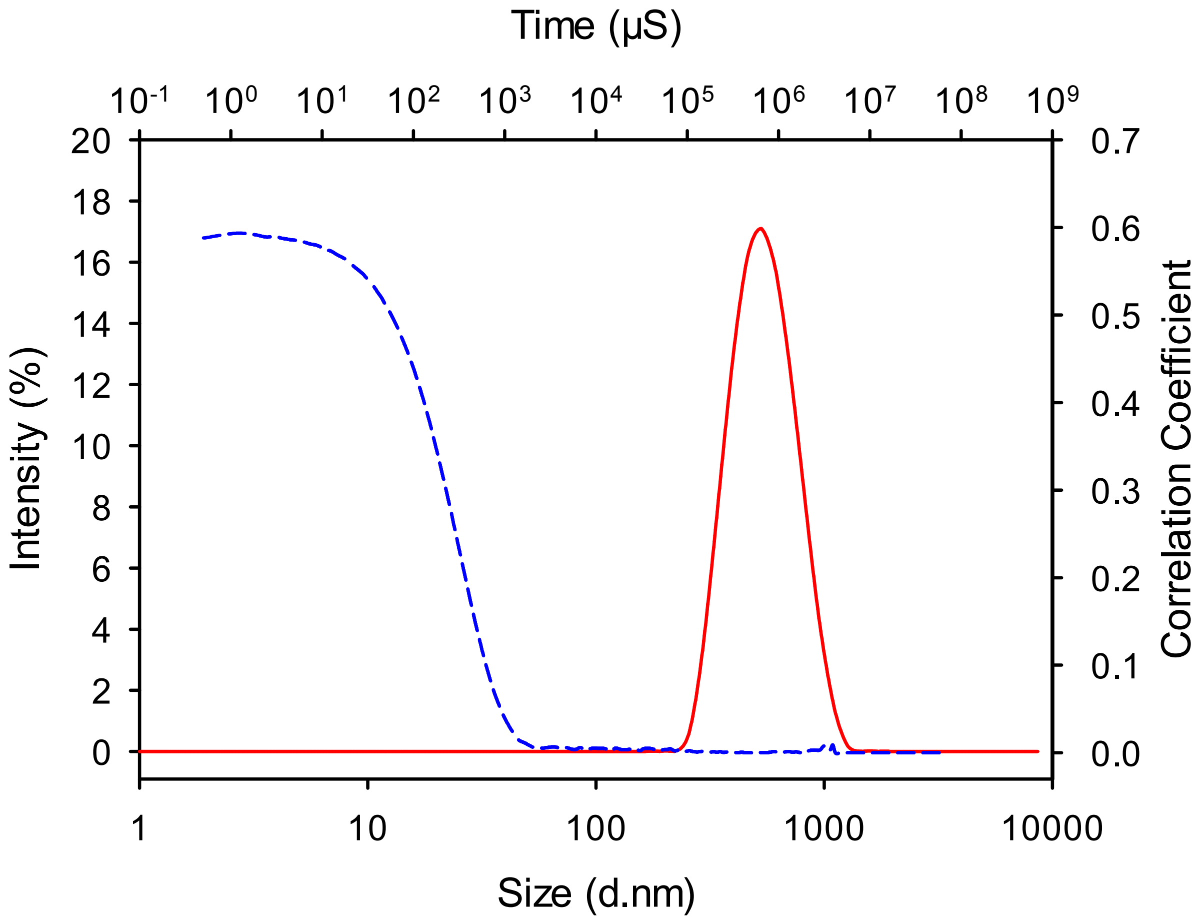


Figure S3. Size distribution and correlation coefficient of TDF precipitated with AOT and measured in DCM continuous phase. Measurements are an average of 3 scans using a Malvern Zetasizer Nano S dynamic light scattering instrument.

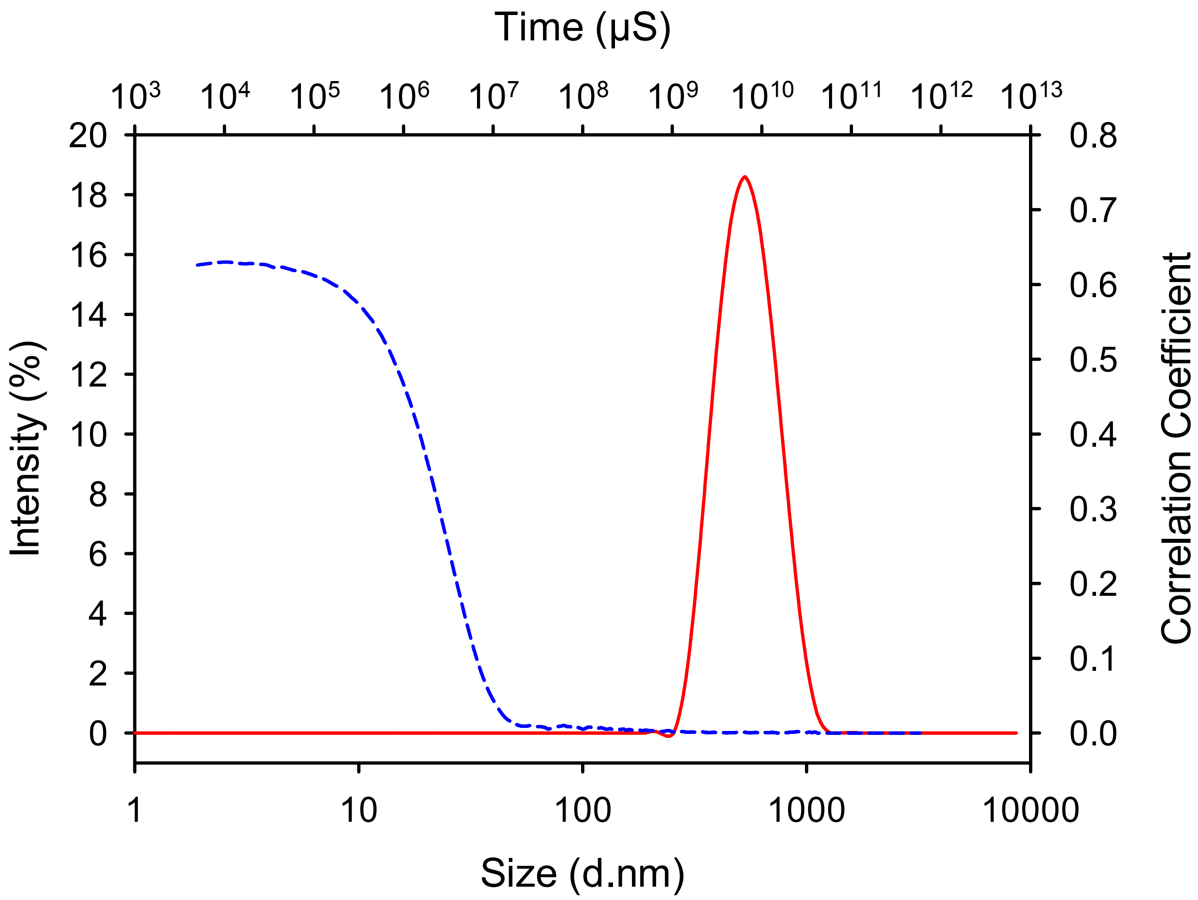


Figure S4. Size distribution and correlation coefficient of TDF precipitated with AOT and Lauroglycol FCC, measured in DCM continuous phase. Measurements are an average of 3 scans using a Malvern Zetasizer Nano S dynamic light scattering instrument.

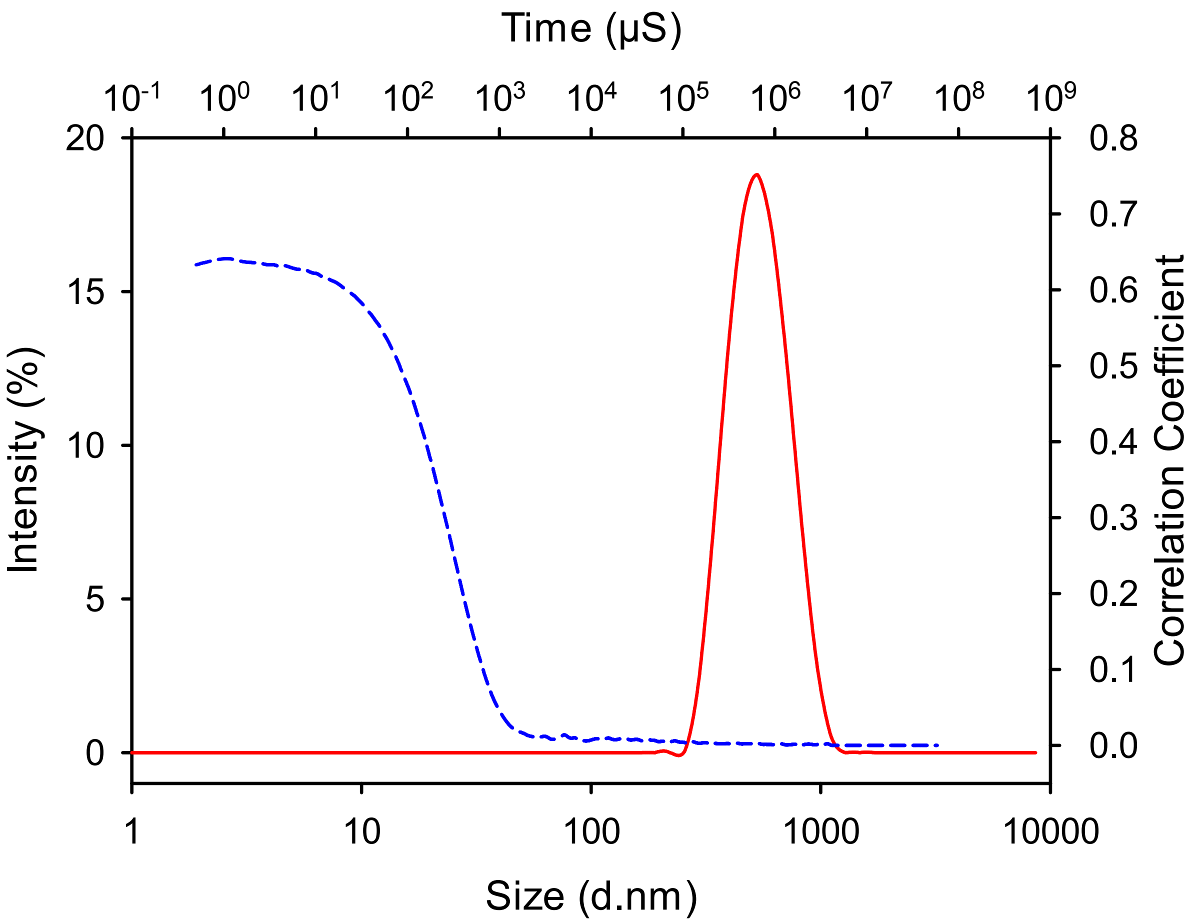


Figure S5. Size distribution and correlation coefficient of TDF precipitated with AOT and Maisine 35-1, measured in DCM continuous phase. Measurements are an average of 3 scans using a Malvern Zetasizer Nano S dynamic light scattering instrument.

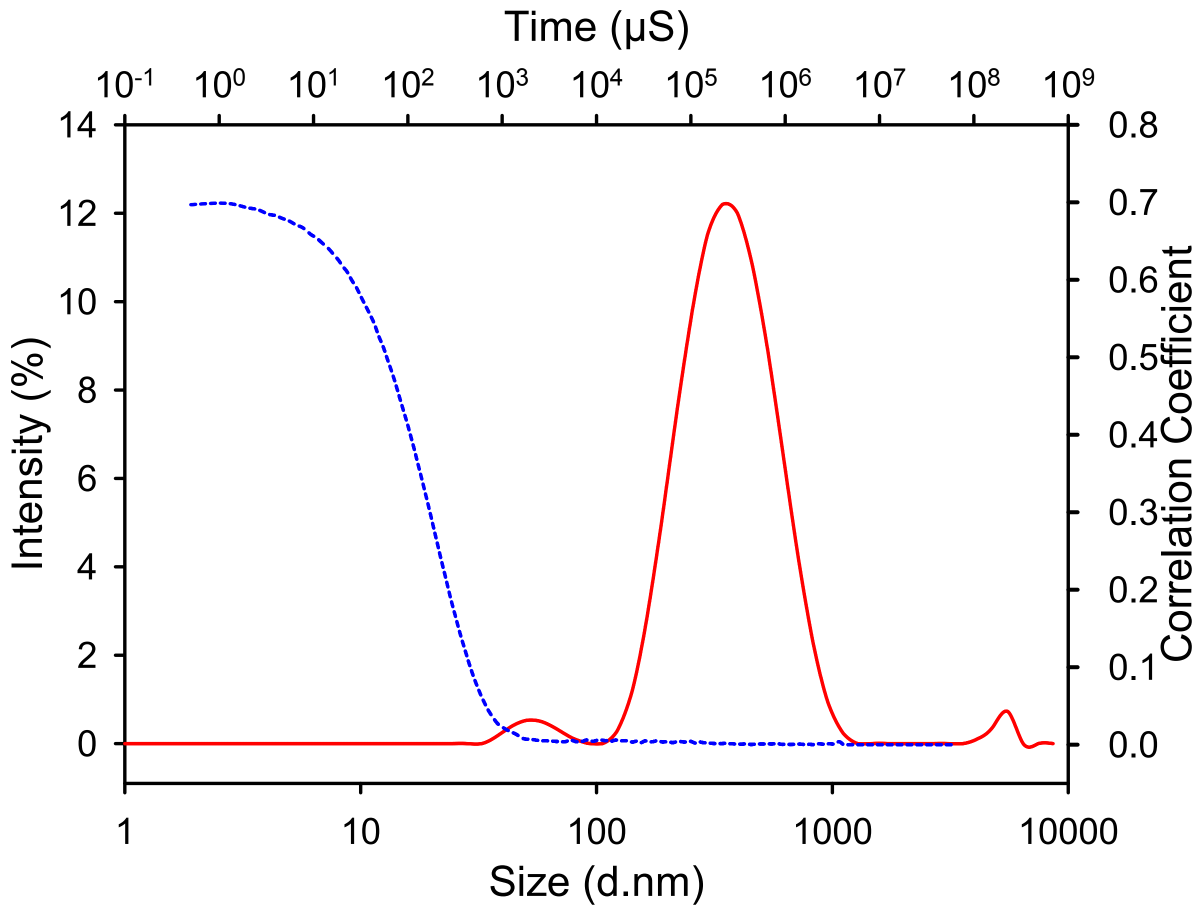


Figure S6. Size distribution and correlation coefficient of TDF precipitated with AOT and then freeze dried in the presence of sesame oil. Particles in oil samples are diluted into DCM prior to measurement. Measurements are an average of 3 scans using a Malvern Zetasizer Nano S dynamic light scattering instrument.

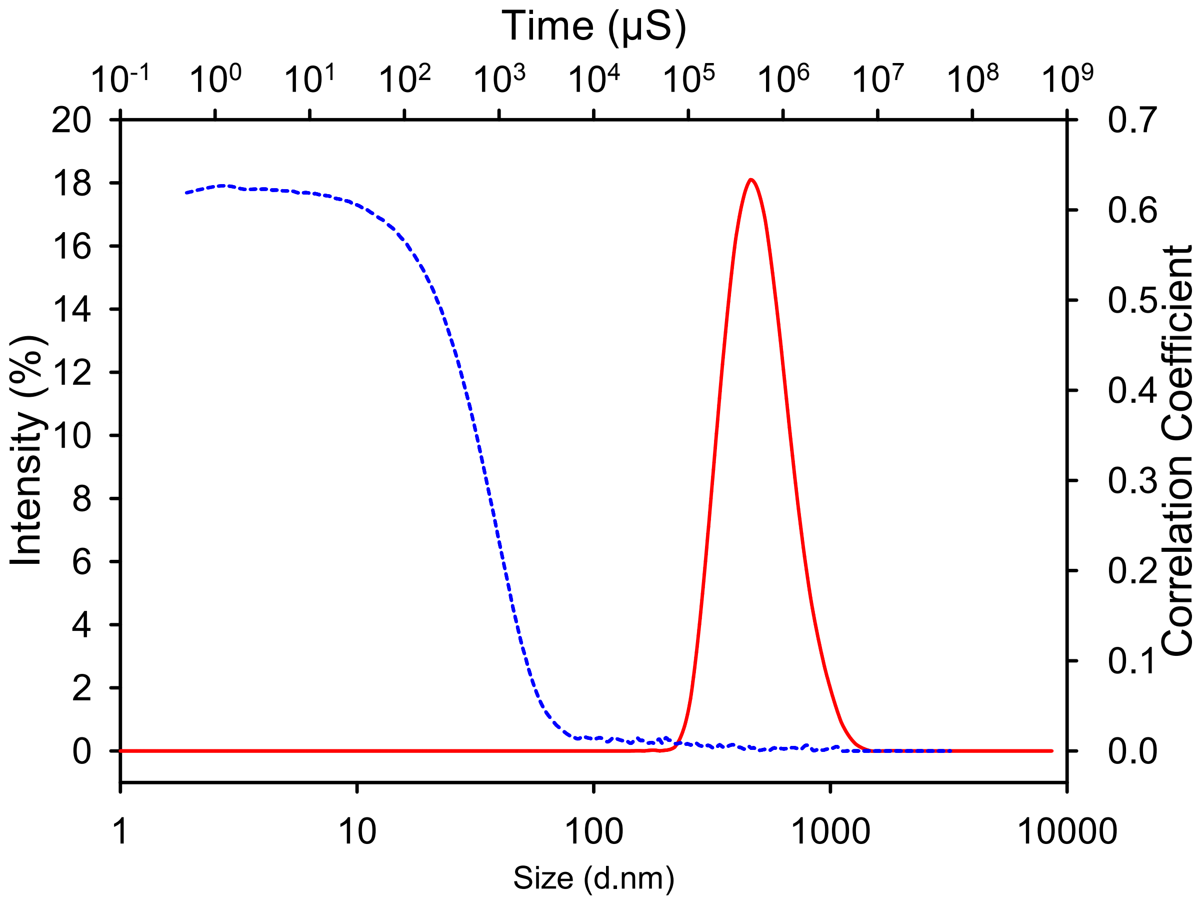


Figure S7. Size distribution and correlation coefficient of TDF precipitated with AOT/Lauroglycol FCC and then freeze dried in the presence of sesame oil. Particles in oil samples are diluted into DCM prior to measurement. Measurements are an average of 3 scans using a Malvern Zetasizer Nano S dynamic light scattering instrument.

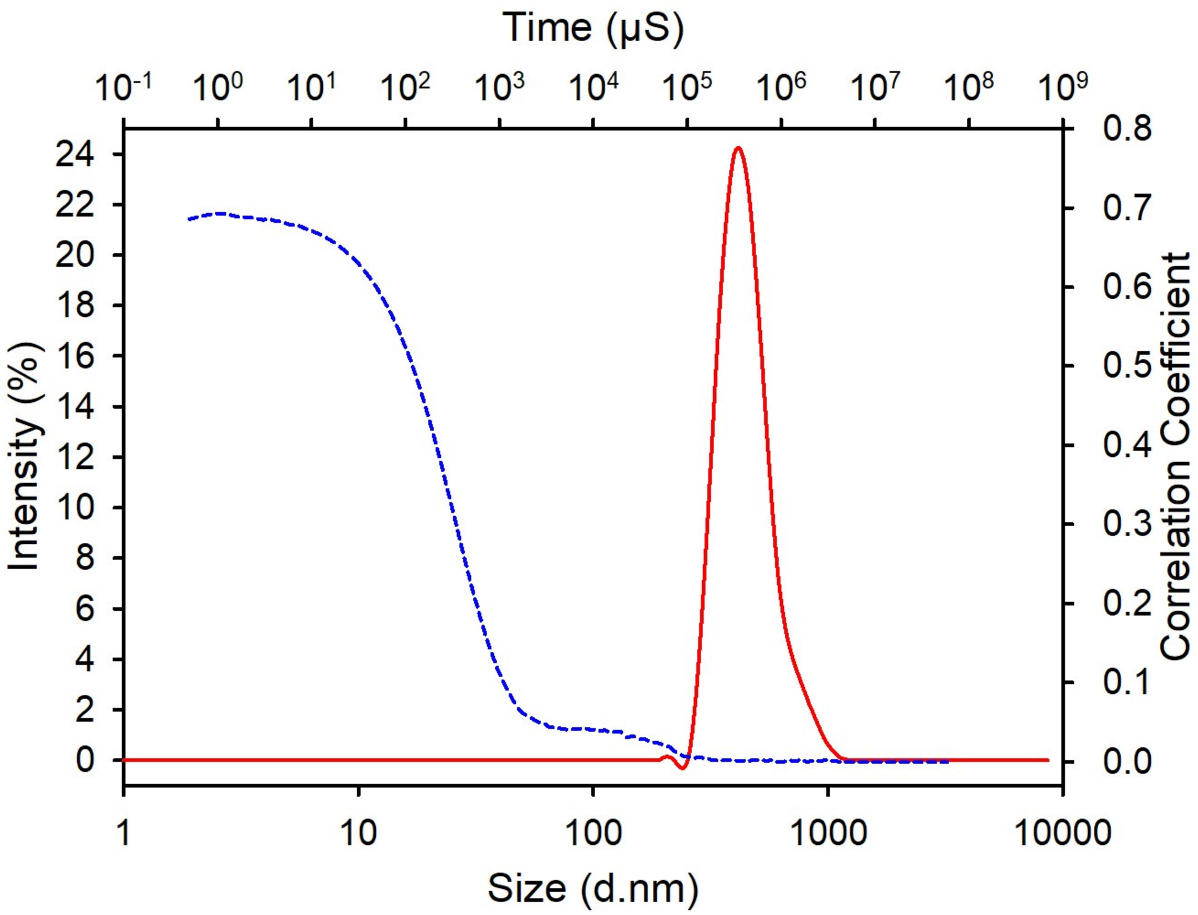


Figure S8. Size distribution and correlation coefficient of TDF precipitated with AOT/Maisine 35-1 and then freeze dried in the presence of sesame oil. Particles in oil samples are diluted into DCM prior to measurement. Measurements are an average of 3 scans using a Malvern Zetasizer Nano S dynamic light scattering instrument.

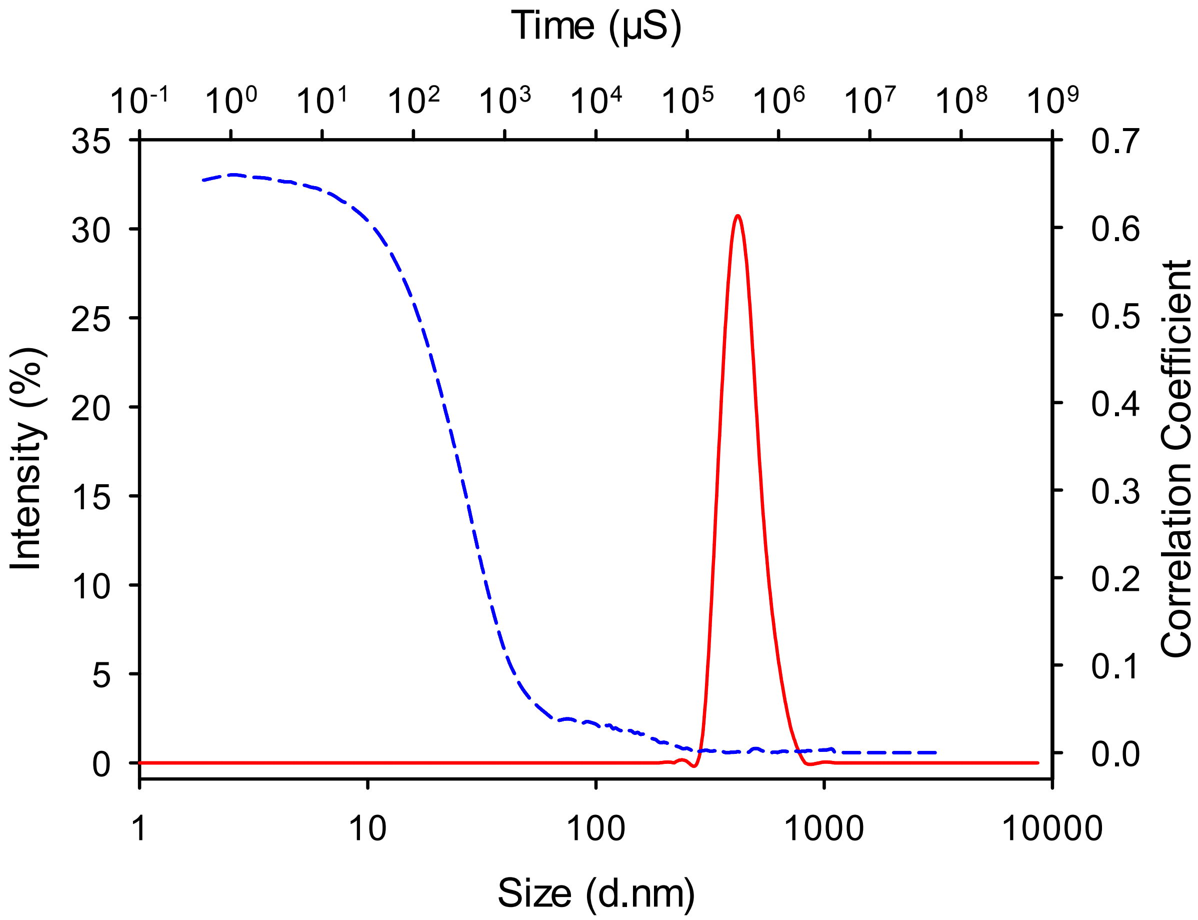


Figure S9. Size distribution and correlation coefficient of TDF precipitated with AOT and then freeze dried in the presence of peanut oil. Particles in oil samples are diluted into DCM prior to measurement. Measurements are an average of 3 scans using a Malvern Zetasizer Nano S dynamic light scattering instrument.

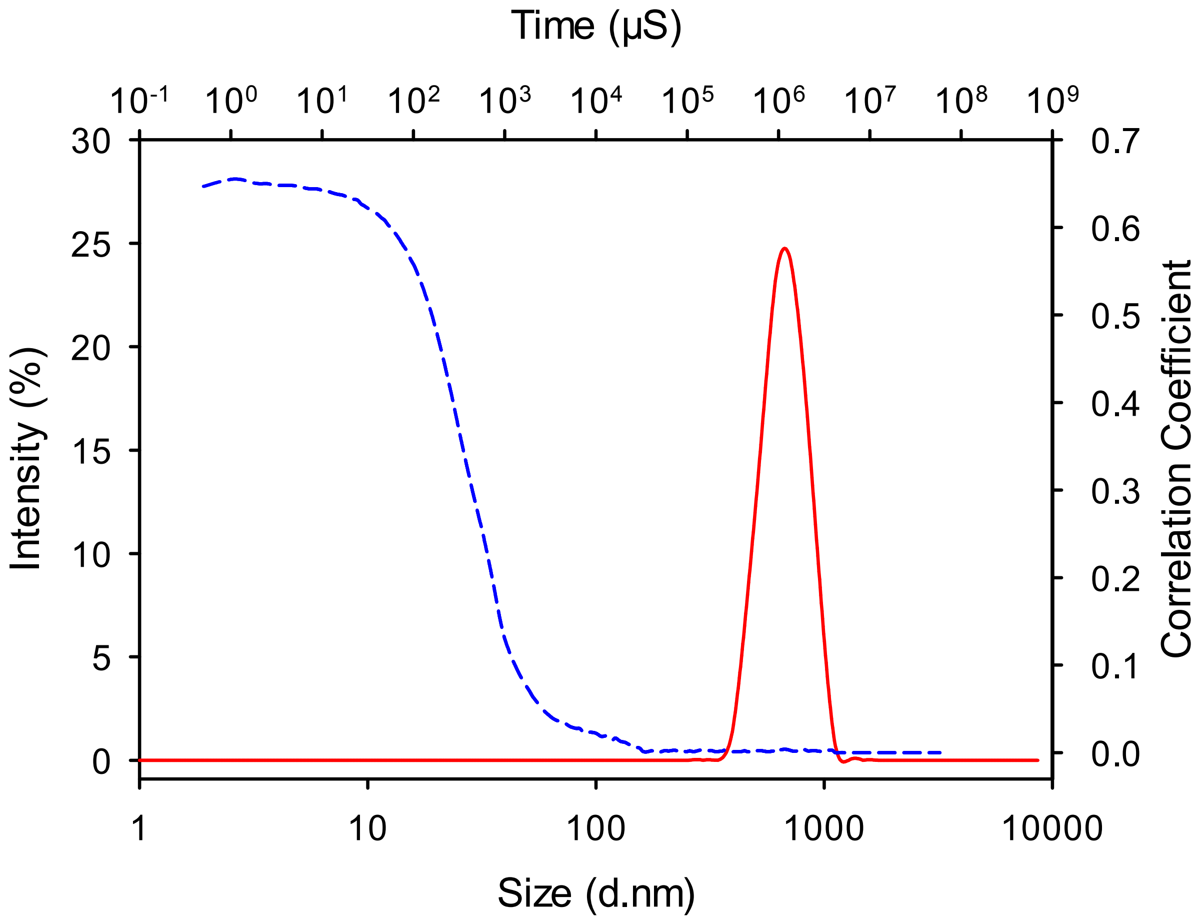


Figure S10. Size distribution and correlation coefficient of TDF precipitated with AOT/Lauroglycol FCC and then freeze dried in the presence of peanut oil. Particles in oil samples are diluted into DCM prior to measurement. Measurements are an average of 3 scans using a Malvern Zetasizer Nano S dynamic light scattering instrument.

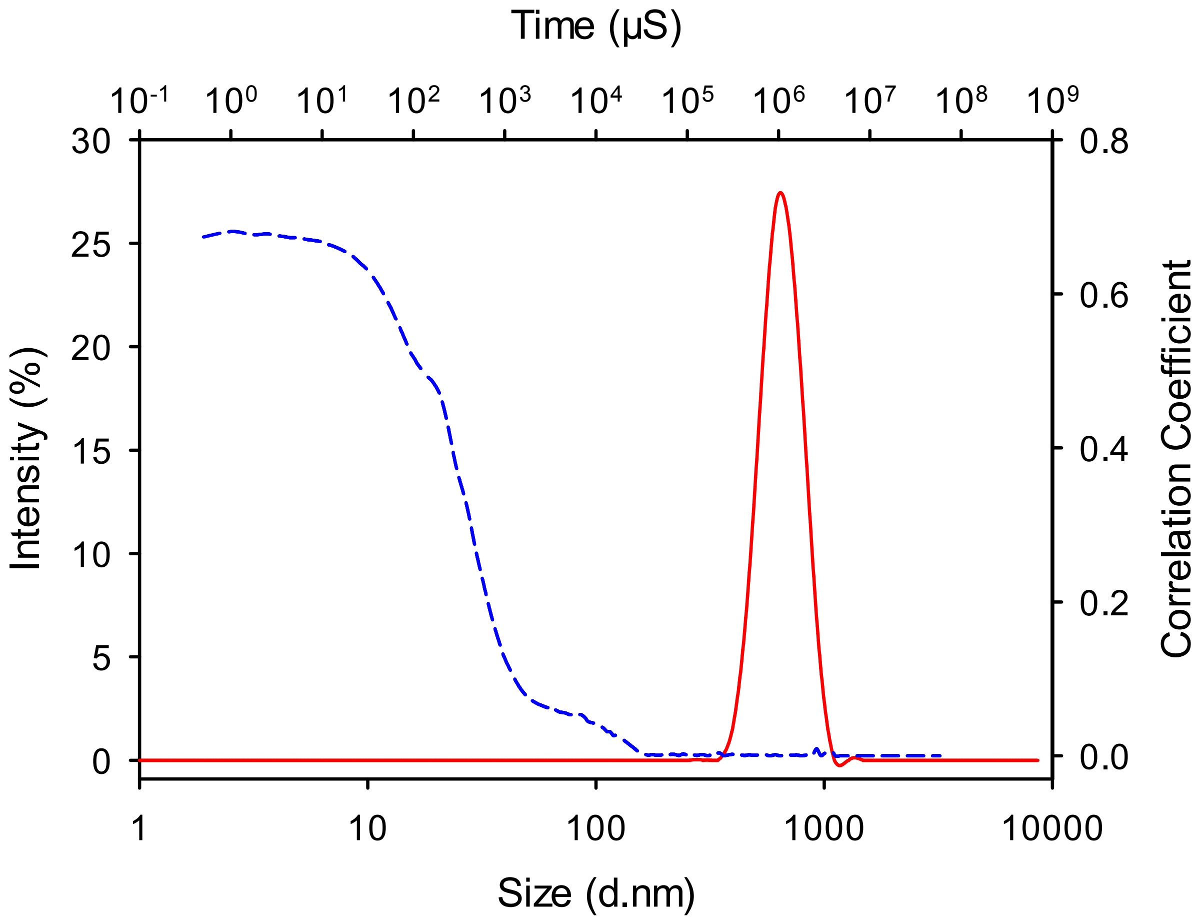


Figure S11. Size distribution and correlation coefficient of TDF precipitated with AOT/Maisine 35-1 and then freeze dried in the presence of peanut oil. Particles in oil samples are diluted into DCM prior to measurement. Measurements are an average of 3 scans using a Malvern Zetasizer Nano S dynamic light scattering instrument.

Table S2: Complete data table from DLS analysis of nanoprecipitates included in this manuscript

Table 1. Dynamic light scattering of varying TDF nanoprecipitates

| Stabiliser | DLS[a] | | | | Derived Count rate | Attenuator |
|---|---|---|---|---|---|---|
| | Dz (nm) | Dn (nm) | Dv (nm) | PDI | | |
| No Oil | | | | | | |
| LG FCC/M 35-1* | *5874* | *55* | *56* | *0.780* | 342 | 10 |
| AOT | 540 | 478 | 629 | 0.164 | 30136 | 7 |
| AOT/LG FCC | 545 | 489 | 617 | 0.148 | 23227 | 7 |
| AOT/M 35-1 | 540 | 485 | 611 | 0.177 | 23040 | 7 |
| Sesame Oil | | | | | | |
| AOT | 295 | 205 | 1443 | 0.373 | 23987 | 7 |
| AOT/LG FCC | 512 | 441 | 572 | 0.242 | 30254 | 7 |
| AOT/M 35-1 | 584 | 421 | 490 | 0.343 | 27693 | 7 |
| Peanut Oil | | | | | | |
| AOT | 672 | 426 | 462 | 0.456 | 26852 | 7 |
| AOT/LG FCC | 840 | 654 | 705 | 0.383 | 21321 | 7 |
| AOT/M 35-1 | 815 | 632 | 680 | 0.327 | 23224 | 7 |

*poor data quality reported for completeness

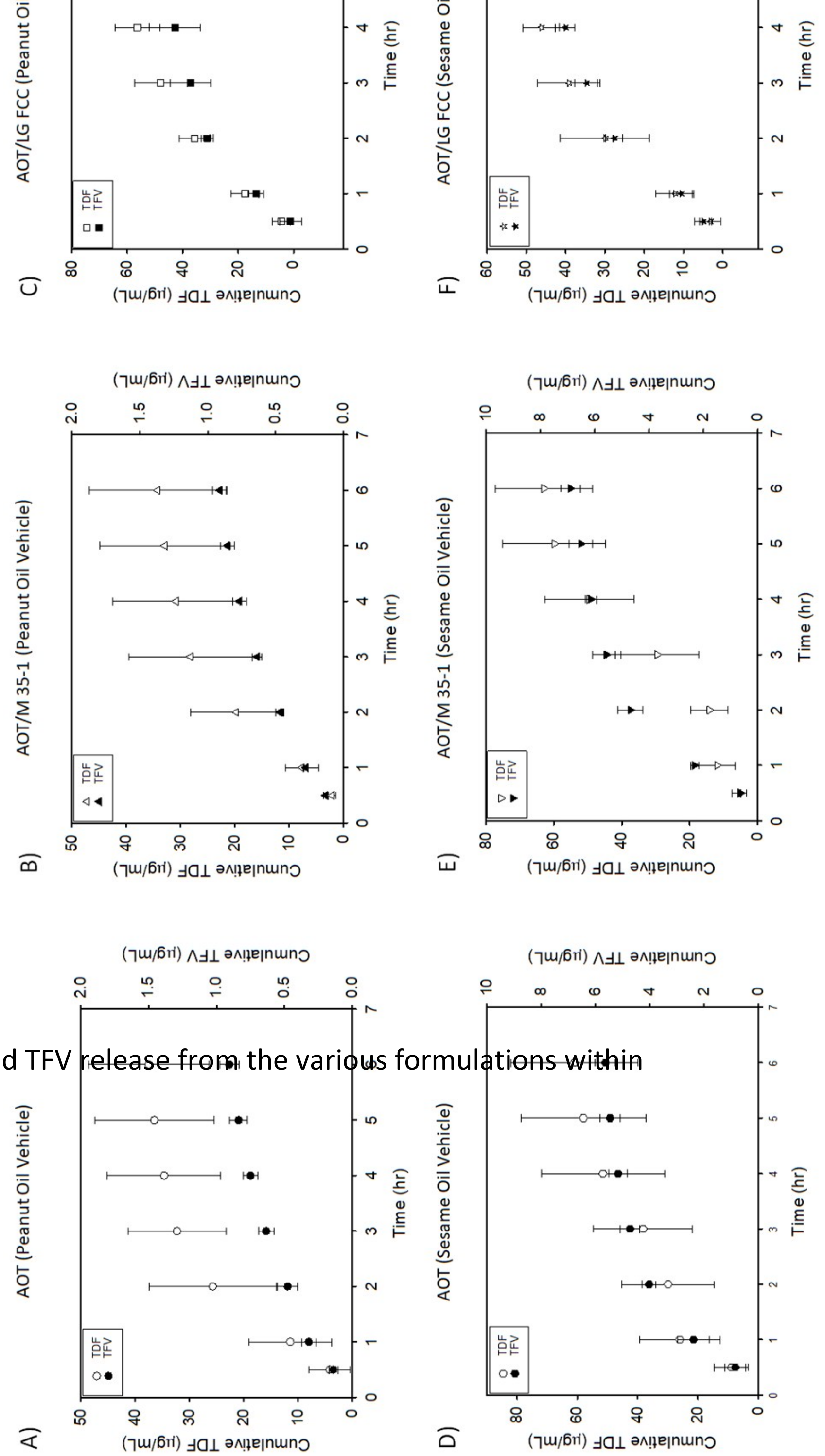


Figure S12. Comparison of TDF and TFV release from the various formulations within different oil vehicles

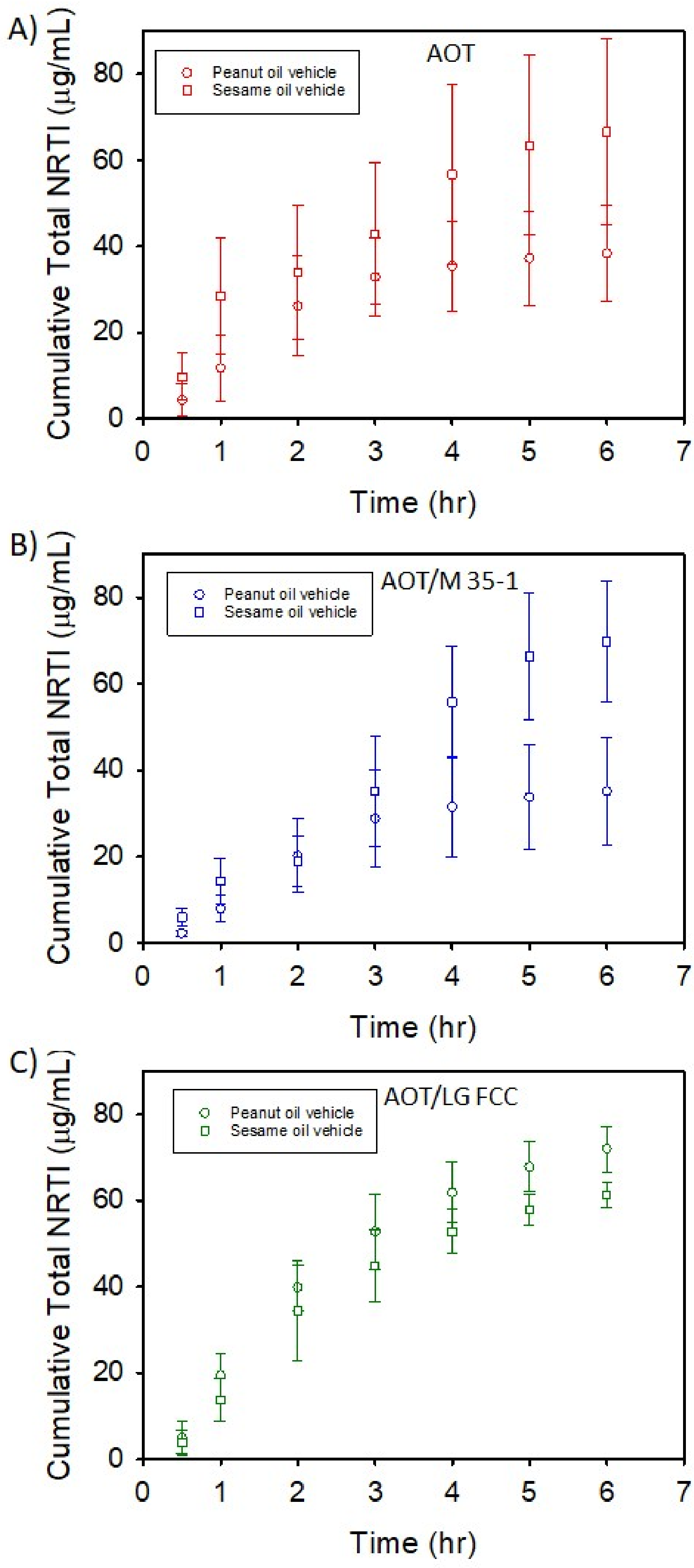


Figure S13. Comparison of total NRTI release from the various formulations within different oil vehicles
